# Lower expression of Bax predicts poor clinical outcome in patients with glioma after curative resection and radiotherapy/chemotherapy

**DOI:** 10.1007/s11060-018-03031-9

**Published:** 2018-11-16

**Authors:** Pei-Guo Wang, Yu-Ting Li, Yi Pan, Zhen-Zhu Gao, Xu-Wen Guan, Li Jia, Feng-Ting Liu

**Affiliations:** 1 Department of Radiotherapy, Key Laboratory of Cancer Prevention and Therapy, National Clinical Research Centre for Cancer, Tianjin Medical University Cancer Institute and Hospital, Tianjin’s Clinical Research Center for Cancer, Tianjin, 300060 China; 2 The Graduate School, Tianjin Medical University, Tianjin, 300070 China; 3 Department of Pathology, Key Laboratory of Cancer Prevention and Therapy, National Clinical Research Centre for Cancer, Tianjin Medical University Cancer Institute and Hospital, Tianjin’s Clinical Research Center for Cancer, Tianjin, 300060 China; 4 Centre for Haemato-Oncology, Barts Cancer Institute, Queen Mary University of London, London, EC1M 6BQ UK

**Keywords:** Bax, Clinical outcome, Glioma, Radiotherapy, And chemotherapy

## Abstract

**Background:**

The prognosis in patients with gliomas after surgical resection followed by radiotherapy and/or chemotherapy is still very poor. The pro-apoptotic protein Bax, a short-lived protein in cancers, plays important roles in the sensitivity of glioma cells to spontaneous and therapy-induced apoptosis but and its prognostic value in gliomas is unknown.

**Methods:**

By an immunohistochemical method, we determined Bax protein expression from 96 patients with gliomas after curative resection. Two statistical analyses were performed to evaluate the prognostic significance of Bax protein: an independent continuous and a multivariate categorical analysis, with test/validation set-defined cut points, and Kaplan–Meier estimated outcome measures of overall survival (OS) and relapse-free survival (RFS).

**Results:**

Bax protein levels in glioblastoma were significantly decreased compared with grade II gliomas. Lower levels of Bax expression confer worse OS (continuous *P* = 0.025; categorical *P* = 0.003) and RFS (continuous *P* = 0.014; categorical *P* < 0.0001) and negatively correlate with the grades of gliomas. Patients underwent radiotherapy followed by surgical resection showed significantly increased OS (median = 45 vs. 17 months) and RFS (median = 39 vs. 16 months). Patients with higher levels of Bax and radiotherapy showed greatly increased survival rates (median OS = 66 months and median RFS = 105 months). Lower expression of Bax also confers inferior clinical outcome for gliomas patients after chemotherapy with temozolomide (OS and RFS P < 0.0001).

**Conclusion:**

Decreased expression of Bax correlates with poor clinical outcome in patients with gliomas. We propose that Bax protein levels can be used as a reliable prognostic marker for risk-stratify patients with gliomas after curative resection and radiotherapy and/or chemotherapy.

**Electronic supplementary material:**

The online version of this article (10.1007/s11060-018-03031-9) contains supplementary material, which is available to authorized users.

## Introduction

Gliomas are the most common type and account for 80% of all malignant primary brain tumors [1]. Under the WHO classification, gliomas are divided into four histological grades [2, 3]. The grade IV gliomas, also called glioblastoma are fast growing, the deadliest and incurable brain cancer compared with the grade I to III gliomas. Despite comprehensive treatment with surgery, radiotherapy, and chemotherapy with temozolomide (TMZ), the prognosis of glioblastoma is poor, with a median survival of 14.5–16.6 months and worse quality of life throughout the disease course [4, 5]. The dismal prognosis conferred by glioblastoma is partly caused by the tendency to diffusely infiltrate into neighboring brain tissue, but also by the inherent resistance of these tumors to both chemotherapy and radiation [6]. However, the mechanisms by which glioblastoma are resistant to conventional therapies are poorly understood.

The Bcl-2 family of proteins plays important role in the regulation of apoptosis. Increased expression of anti-apoptotic Bcl-2 or decreased expression of pro-apoptotic Bax confers resistance of tumor cells to apoptosis in many cancers [7]. It was recently reported that combined treatment with metformin and TMZ overcomes resistance of glioblastoma cells to apoptosis by increasing the ratio of Bax/Bcl-2 [8]. Bax-deficient glioblastoma cells are highly resistance to various apoptotic stimuli compared with Bax-expressing glioblastoma cells [9]. Activation or overexpression of Bax in glioblastoma increases the sensitivity of these cells to apoptosis [10, 11]. Several human tumor tissues, such as human pancreatic cancer [12], breast cancer [13], chronic lymphocytic leukemia [14], colorectal cancer [15, 16] and non-small cell lung cancer [17], have decreased expression of Bax protein which is significantly associated with poor clinical outcomes of these cancers. It has been proposed that modulation of Bax expression may be a useful therapeutic modality for gliomas [11, 18].

Bax is a short-lived protein in cancer cells and its degradation is ubiquitin/proteasome-dependent [14, 19]. We previously reported that increased Bax degradation activity is associated with worse clinical outcome in human chronic lymphocytic leukemia [14]. The levels of Bax protein expression in human gliomas tissue and its impact on the clinical outcomes in patients with gliomas are highly controversy due to limited case numbers [9, 20–23]. It was recently reported that the intratumoral administration of proteasome inhibitor bortezomib into the cranial cavity significantly increased survival rate of glioma-bearing mice [24], suggesting that the ubiquitin/proteasome system plays an important role in the disease progression of gliomas.

In this study, we aimed to determine whether levels of Bax protein expression have impact on clinical outcomes in 50 patients with glioblastoma and 46 patients with grade II or III gliomas. The levels of Bax mRNA and protein expression in glioblastoma and normal brain were analyzed to explore the effect of Bax instability in patients with glioblastoma. We also provide clinical data of the impact of Bax levels on the efficacy of radiotherapy and chemotherapy in patients with gliomas.

## Materials and methods

### Patients and samples

Formalin-fixed, paraffin-embedded gliomas tissue specimens were randomly selected from the archives of the Department of Pathology of the Tianjin Medical University Cancer Institute and Hospital (Tianjin, China). Samples used were from 96 glioma patients, who underwent initial surgical resection between December 2006 and September 2016 in Tianjin Cancer Hospital. Ethical approval for this study was obtained from the local ethics committee. Patients gave verbal consent for the use of their tumor tissues for future investigations, which had been performed for many years at time of the initial diagnosis. All specimens were handled and made anonymous according to ethical and legal standards. None of the patients had received radiotherapy or chemotherapy prior to surgery. The patient demographics and clinical characteristics are summarized in Supplementary Table 1. The diagnosis of these glioma samples was verified by two senior pathologists according to the 2016 WHO classification of tumors of the central nervous system [2, 3]. Detailed pathologic and clinical data were collected for all samples, including the WHO grade. This cohort includes 50 WHO IV, 24 WHO III and 22 WHO II glioma patients. The median follow-up time for overall survival (OS) and relapse-free survival (RFS) were 28 and 22.6 months, respectively (Supplementary Fig. 1; Supplementary Tables 1 and 2). In addition, 21 normal brain control tissues were obtained from patients who underwent surgery for non-neoplastic tissues adjacent to the tumor. Comparison of Bax mRNA expression levels between normal brain (n = 10) and glioblastoma (n = 542 cases) was obtained from The Cancer Genome Atlas (TCGA) brain dataset, Oncomine (https://www.oncomine.org/). Two datasets (n = 206, and n = 151 cases, respectively) for analyzing the association of Bax mRNA levels with clinical outcomes in patients with multiform glioblastoma were obtained from TCGA database [25, 26].

### Follow-up and primary endpoints

A repeat tumor-imaging study was performed approximately 3 months after resection or completion of radiotherapy. Postoperative patients were followed-up periodically to exclude relapse of glioma. Update inquiries about current survival status of all patients were made by telephone calls periodically by the end of April 2017. Recurrence or progression was declared mainly on the basis of magnetic resonance imaging (MRI), the second postoperative pathologic reports according to the WHO guidelines, and the comprehensive diagnosis of Multidisciplinary Team (MDT). Team doctors from departments of neurosurgery, pathology, radiology and radiobiology working together to diagnose relapse according to the Response Assessment in Neuro-Oncology Criteria (RANO) to exclude pseudo-progression [27, 28]. The co-primary endpoints were the duration of OS from randomization, which was defined as the diagnosis time until death from any cause, and the duration of RFS, which was defined as the diagnosis time until either disease progression or death [4].

### Immunohistochemistry (IHC)

Glioma specimens were formalin fixed, paraffin embedded and sectioned (5 µm in thickness). The slides were de-paraffinized in xylene and rehydrated through graded ethanol to water before staining. All sections were treated with 5 mM citrate buffer (pH 6.0) for antigen retrieval and with 3% H_2_O_2_ for the inactivation of endogenous peroxidase. After blocking for 30 min, sections were incubated with a mouse anti-Bax antibody (Santa Cruz Biotechnology, sc-7480, 1:200 dilution) overnight at 4 °C. After wash, the sections were stained with a secondary antibody for 30 min at room temperature. Diaminobenzidine and hematoxylin were used as a chromogen substrate and for nuclear counterstaining, respectively [29].

### Evaluation by IHC

The whole tissue fields (× 400 and × 200) were examined under a light microscope. Immunoreactivity for Bax proteins was scored by evaluating the number of positive tumor cells over the total number of tumor cells, the intensity of tumor cells was scored according to morphological criteria. Scores were assigned by using 5% increments (0%, 5%, 10% …100%). Expression for Bax was independently assessed by two pathologists, who were blinded to the clinicopathological data. Their conclusions agreed in approximately 90% of the cases, indicating that this scoring method is highly reproducible. If two assessments were consistent with the results they scored, the value was selected. In cases where completely different results occurred, the appropriate score was further discussed and agreed by two assessors.

### Statistical analysis

Statistical analysis was performed using IBM SPSS version 19.0 for Windows and GraphPad Prism version 5.01. To increase the robustness of statistical inferences, two independent analyses were performed: categorical and continuous data analysis. The categorical data analysis was a clinically applicable method, dividing patients categorically into two groups on the basis of levels of Bax expression (high and low). Categorical (cut point) data analysis was performed using the X-tile statistical package (Yale University, New Haven, CT, USA) [30]. X-Tile divides the cohort into two independent data sets (a test set and a validation set) in a 1:2 ratio, determines optimal cut points for each marker for the test set, and applies this to the validation set [29, 31]. The continuous data analysis was performed as continuous variables by using Cox regression analysis to value the prognostic effect of the biomarker.

Both unpaired *t* test and analysis of variance (ANOVA) test were performed for group comparisons, and correlations between two variables were evaluated using Spearman’s rank correlation test. The chi-squared test is used to determine whether there is a significant difference between the expected frequencies and the observed frequencies in Bax expression and other more categories risk factors.

Kaplan–Meier curves defined by the cut points were generated, according to the P values, hazard ratio (HR) and 95% confidence interval (CI) determined by log-rank test. Univariate analyses were performed first, including one factor a time to examine their prognostic effects. Multivariate analysis was performed using a Cox proportional hazards model to define the independent effect of prognostic variable. Relevant factors were considered simultaneously through the use of enter stepwise models. After the continuous and categorical variables were adjusted, the confounding effect between two risk factors were calculated. The estimated measures of univariate analysis before (HR_crude_) and multivariate analysis after adjusting (HR_adjusted_) for confounding were calculated as: magnitude of confounding = (HR_crude_ − HR_adjusted_)/HR_crude_ × 100%. If the difference between the two measures of association is 10% or more, then confounding was present [29] (http://sphweb.bumc.bu.edu). All P values less than 0.05 were considered statistically significant. *, ** and *** indicate P value < 0.05, 0.01 and 0.001, respectively [29, 32].

## Results

### Bax protein expression is significantly decreased in glioblastoma compared with low grade gliomas

**Fig. 1:**
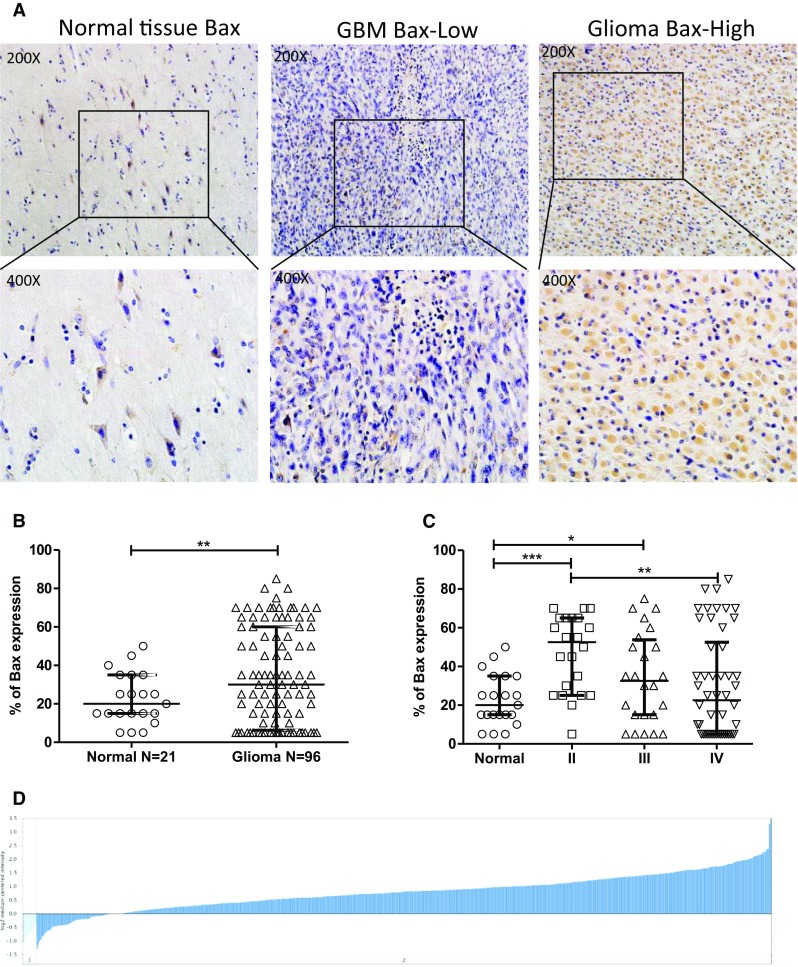
Comparison of levels of Bax protein and Bax mRNA expression in glioma specimens. **a** Representative examples of control, low and high expression of Bax protein expression in Gliomas. Bax expression is shown by horseradish peroxidase-diaminobenzidine (HRP-DAB) immunostaining. **b** The levels of Bax protein expression were presented as % of Bax positive cells. Data presented are medians with interquartile ranges. **c** Data presented are medians of Bax protein expression with interquartile ranges: 52.5% (25–65%) for WHO II grade, 32.5% (15–55%) for WHO III grade and 22.5% (5–50%) for WHO IV grade glioma tissues. The significant difference in Bax protein expression between glioma and normal brain tissues were analysed using unpaired *t* test. **d** Bax mRNA levels in 10 normal Brain tissues and 542 GBM tissues were performed by TCGA brain statistics from http://www.oncomine.org. The P value (1.39E-10) of Bax mRNA levels between normal and GBM are significantly different

Upregulation and activation of Bax is a crucial step for treatment-induced apoptosis in glioblastoma [10, 11, 33]. However, Bax expression status in human glioma is still elusive. To compare the levels of Bax in different grade of gliomas, expression of Bax protein was determined in 96 gliomas and 21 normal brain control tissues using IHC. Bax expression was detected in both glioma and normal brain tissues (Fig. 1a). The levels of Bax were expressed as % of Bax positive cells. Bax expression in glioma samples displayed a heterogeneous expression pattern and the median levels (35%) were significantly higher than in the non-malignant normal controls (23%; P < 0.01; Fig. 1b). Further stratification showed that the grade II and III glioma have significantly increased Bax expression compared with the control (P < 0.001; P < 0.05; Fig. 1c). However, the grade IV (glioblastoma) showed significantly decreased Bax protein expression compared with the grade II tumors (29.70 ± 3.76 vs. 46.82 ± 4.22; P < 0.01; Fig. 1c). Bax mRNA levels in glioblastoma are significantly greater than in the normal brain control tissues (Fig. 1d). However, our data showed that Bax protein levels in glioblastoma was not higher than the control. These results demonstrate that Bax protein expression increases in the early stage of glioma but decreases with disease progression towards the late stage. This may be, at least partly, due to increased Bax degradation activity in glioblastoma.

### Continuous data analysis for the prognostic significance of Bax protein levels in patients with gliomas

The prognostic significance of Bax on the clinical outcomes in glioma patients was first determined using continuous data analysis and compared with other risk factors. Bax expression showed significant impact on OS (P = 0.025; HR = 0.987) and RFS (P = 0.014; HR = 0.984). Other risk factors (continuous variables), such as age and WHO grade also showed prognostic significance on OS and RFS (Table 1). Not surprising, the hazard ratios (HRs) of both age and WHO grade were higher than 1, meaning older age or higher WHO grade patients had higher risk. However, HRs of Bax in both OS and RFS were lower than 1, indicating that lower levels of Bax were associated with poor prognosis. For determining whether Bax was an independent prognostic marker, multivariate analysis was then conducted by combination of Bax with age and WHO grade. The HRs of Bax still remained lower than 1, indicating Bax was an independent prognostic marker for patients with glioma. Although the confounding effect exists between WHO grade and other factors, the prognostic significance of WHO grade on both OS and RFS retained after adjusting (Table 1 and Supplementary Table 3). These results demonstrate that Bax as well as WHO grade are independent prognostic biomarkers for predicting the clinical outcomes in patients with gliomas.

**Table 1:** Uni- and multi-variate analysis of continuous covariates of OS and RFS

Outcome	Covariates	Univariate analysis		Multivariate analysis	
		HR (95% CI)	*P*	HR (95% CI)	*P*
OS	Bax	0.987 (0.975–0.998)	0.025	0.992 (0.982–1.003)	0.160
	Age	1.026 (1.007–1.046)	0.009	1.013 (0.991–1.036)	0.263
	WHO Grade	2.765 (1.802–4.241)	< 0.0001	2.476 (1.577–3.886)	< 0.0001
RFS	Bax	0.984 (0.971–0.997)	0.014	0.991 (0.979–1.003)	0.127
	Age	1.030 (1.008–1.053)	0.008	1.021 (0.995–1.049)	0.115
	WHO Grade	3.670 (2.205–6.109)	< 0.0001	3.288 (1.947–5.553)	< 0.0001

Covariates that were included in the multivariate analysis were selected using the Enter stepwise

### Categorical data analysis for the prognostic significance of Bax protein levels in patients with gliomas

We next used categorical data analysis to determine the association between Bax levels and clinical outcomes. Two variables with low and high expression of Bax were defined using the X-Tile software to generate cut points for each outcome. Age was categorized by median age. WHO grades were categorized as grade II, III and IV groups. The radiotherapy or chemotherapy for patients were also categorized as with (Yes) or without (No) groups (Table 2 and Supplementary Table 4). Chi square distribution analysis showed that 95.5% grade II, 79.2% grade III and 58.2% grade IV glioma patients express higher Bax in (P < 0.003) and 68.8% relapsed patients vs. 94.4% non-relapsed patients express higher Bax (P < 0.027). Interestingly, 82.5% tumors in the frontal lobe express higher Bax vs. 57.1% tumors vs. in the temporal lobe (P < 0.032 and Supplementary Table 4). Log rank analysis showed that higher expression of Bax was significantly associated longer OS with median OS for Bax^HIGH^ = 44.62 and Bax^LOW^ = 16.95 months (P = 0.0003; HR = 0.365) and longer RFS with median RFS for Bax^HIGH^ = 50.43 and Bax^LOW^ = 8.8 months (P < 0.0001; HR = 0.312) (Table 2). Other categorical risk factors, such as age, gender (female vs. male), tumor numbers (multiple vs. single), tumor location (temporal vs. frontal), relapse (yes vs. no), WHO grade (IV vs. III vs. II) all showed prognostic significance on both OS and RFS (Table 2; Supplementary Figs. 2, 3 and 4). Bax mRNA expression in glioblastoma and clinical outcomes of two independent cohorts [25, 26] was analyzed retrospectively (online accessible TCGA database). The levels of low and high mRNA of Bax were also defined by the X-Tile software to generate cut-off points for each outcome. Bax mRNA levels in glioblastoma patients from two datasets did not show any prognostic significance on both OS and RFS (Supplementary Fig. 5; Supplementary Table 5). Similarly, Bax protein levels in glioblastoma patients from our data did not show statistical significance on OS and RFS (Supplementary Fig. 6; Supplementary Table 6). However, the median RFS time in Bax lower expression group was shorter than the higher group (6.4 vs. 10.2 months). These results indicate that lower expression of Bax protein (mainly in glioblastoma) but not Bax mRNA is significantly associated with poor prognosis in patients with gliomas.

**Table 2:** Uni- and multi-variate analysis of categorical covariates of OS and RFS

Outcome	Covariates (cut points)	OS months^a^	Univariate analysis		Multivariate analysis	
			HR (95% CI)	*P*	HR (95% CI)	*P*
OS	Bax^b^ (> 10 vs. ≤ 10)	45:17	0.403 (0.220–0.739)	0.0024	0.474 (0.176–1.277)	0.140
	Age^c^ (> 53 vs. ≤ 53)	20:45	1.785 (1.031–3.089)	0.039	1.208 (0.512–2.848)	0.666
	Gender (female vs. male)	23:66	2.001 (1.148–3.490)	0.014	1.479 (0.648–3.374)	0.352
	Tumor number (multiple vs. single)	17:36	1.673 (0.934–2.997)	0.083	0.858 (0.333–2.207)	0.750
	Tumor location (temporal vs. frontal)	19:111	2.440 (1.212–4.911)	0.012	0.639 (0.243–1.682)	0.365
	Relapse (yes vs. no)	25:-	37.807 (2.251–635.0)	0.012	219975 (0–2.91E154)	0.944
	Radiotherapy (yes vs. no)	45:17	0.431 (0.246–0.755)	0.0003	0.409 (0.166–1.007)	0.052
	Chemotherapy (yes vs. no)	28:21	0.801 (0.433–1.480)	0.479	0.661 (0.228–1.917)	0.446
	WHO grade (IV vs. III vs. II)	17:36:112	2.765 (1.802–4.241)	< 0.0001	2.861 (1.396–5.864)	0.004
RFS	Bax^b^ (> 15 vs. ≤ 15)	50:9	0.312 (0.162–0.601)	< 0.0001	0.506 (0.192–1.338)	0.170
	Age^c^ (> 53 vs. ≤ 53)	12:33	1.838 (1.002–3.373)	0.049	1.258 (0.514–3.078)	0.614
	Gender (female vs. male)	16:50	2.041 (1.116–3.732)	0.021	1.451 (0.635–3.315)	0.377
	Tumor number (multiple vs. single)	12:33	1.896 (0.994–3.619)	0.052	0.891 (0.345–2.304)	0.812
	Tumor location (temporal vs. frontal)	10:50	3.090 (1.392–6.855)	0.006	0.702 (0.278–1.773)	0.454
	Relapse (yes vs. no)	13:–	47.505 (3.230–698.6)	0.005	200877 (0–3.14E150)	0.943
	Radiotherapy (yes vs. no)	39:16	0.487 (0.263–0.904)	0.023	0.401 (0.161–0.996)	0.049
	Chemotherapy (yes vs. no)	18:50	1.367 (0.632–2.959)	0.427	0.618 (0.210–1.814)	0.381
	WHO grade (IV vs. III vs. II)	8:29:105	3.670 (2.205–6.109)	< 0.0001	2.894 (1397–5.992)	0.004

Covariates that were included in the multivariate analysis were first selected using the Enter stepwise

^a^Median survival rate (months)

^b^Categorical cut-off points were defined by the X-tile software

^c^Median age

For determining the confounding effect of Bax with other categorical risk factors, Cox regression analysis was then conducted by combination of Bax with multivariable (risk factors) (Table 2). After Bax was adjusted with other prognostic markers, the HRs remained less than one, suggesting no confounding effects between Bax expression and other risk factors. However, Bax levels on prognosis were no longer significant (Table 2). This was due to lower but not higher Bax expression was significantly associated with higher chances of relapse, without radiotherapy and higher grade of glioma. These results further demonstrate that lower levels of Bax protein expression is significantly associated with relapse, higher WHO grade, and shorter survival rates in patients with glioma.

### Higher level of Bax is significantly associated with longer survival rates in glioma patients underwent radiotherapy and/or chemotherapy

**Fig. 2:**
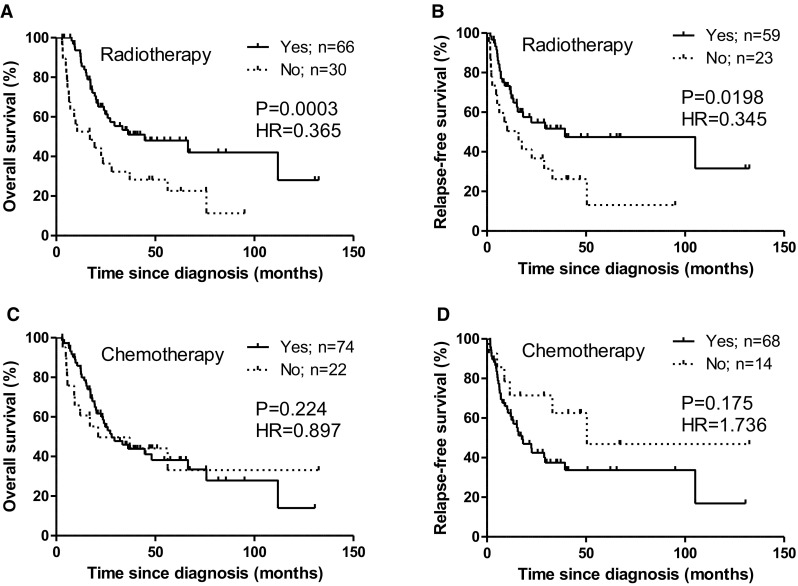
Prognostic effects of radiotherapy (**a, b**) or chemotherapy (**c, d**) on patients with glioma. Kaplan–Meier survival curves of glioma patients according to with or without radiotherapy or chemotherapy were generated with GraphPad Prism program. **a** OS of glioma patients was based on Yes and No. **b** RFS of glioma patients was based on Yes and No. **a, b** ‘Yes’: the patients had completed the radiotherapy with or without chemotherapy. ‘No’: the patients had not undergone or completed the radiotherapy with or without chemotherapy. **c** OS of glioma patients was based on Yes and No. **d** RFS of glioma patients was based on Yes and No. **c, d** ‘Yes’: the patients had chemotherapy with TMZ with or without radiotherapy. ‘No’: the patients had not undergone chemotherapy with or without radiotherapy. ‘n’: numbers of patients

Curative resection, radiotherapy and concomitant chemotherapy with TMZ is the standard treatment approach for glioma patients [34–36]. In this cohort, patients underwent radiotherapy have significantly better clinical outcomes OS (P = 0.003, HR = 0.431) and RFS (P = 0.023, HR = 0.487), determined using Cox regression analysis (Table 2). Kaplan–Meier curves show that the patients completed radiotherapy had a significantly longer OS and RFS compared with the patients had not undergone or completed radiotherapy (Fig. 2a, b). The median OS rate for patients with radiotherapy is 44.62 months vs. 16.95 months for patients without radiotherapy and the median RFS rate for patient with radiotherapy is 39.36 months vs. 15.97 months for patients without radiotherapy. However, patients treated with or without TMZ did not show statistical differences on both OS and RFS (Fig. 2c, d).

**Fig. 3:**
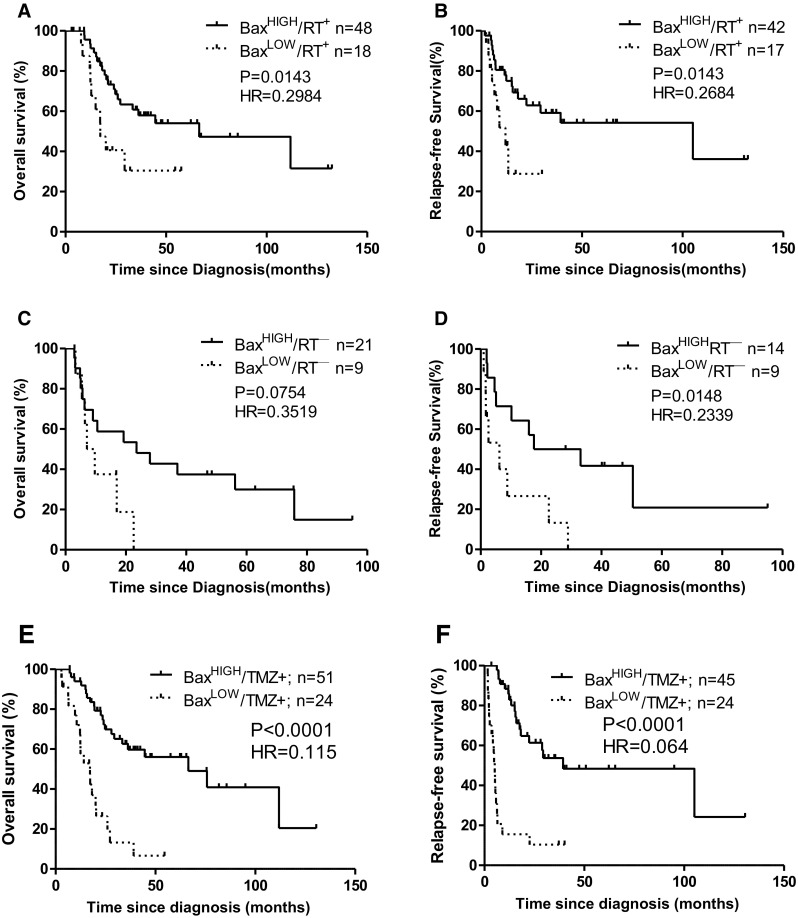
Association between Bax expression and radiotherapy/chemotherapy in clinical outcomes in patients with gliomas. **a, c** Overall survival for patients with or without radiotherapy. Two subgroups were defined according to the cut-off point for Bax (10%) and whether the patients had finished the radiotherapy: Bax^HIGH^/RT^+^ = Bax > 10% and the patients had completed the radiotherapy; Bax^LOW^/RT^−^ = Bax ≤ 10% and the patients had not underwent or completed the radiotherapy. **b, d** Relapse-free survival for patients with or without radiotherapy. Four subgroups were defined according to the cut-off point for Bax (15%) and whether the patients had finished the radiotherapy: Bax^HIGH^/RT^+^ = Bax > 15% and the patients had finished the radiotherapy; Bax^LOW^/RT^−^ = Bax ≤ 15% and the patients had not finished the radiotherapy. **e** Overall survival for patients underwent chemotherapy. The Bax^HIGH^ and Bax^LOW^ groups for OS were defined according to the cut-off point for Bax (10%). **f** Relapse-free survival for patients underwent chemotherapy. The Bax^HIGH^ and Bax^LOW^ groups for RFS were defined according to the cut-off point for Bax (15%). Cut points were generated by the X-Tile software. ‘n’: numbers of patients. TMZ indicates temozolomide

As shown above, Bax protein expression and radiotherapy both are independent risk factors for gliomas (Table 2). We then further determined the association between Bax expression and radiotherapy or chemotherapy on clinical outcomes in patients with gliomas. We combined Bax expression and radiotherapy variables and categorized them into four subgroups: i.e., Bax^HIGH^/RT^+^, Bax^LOW^/RT^+^, Bax^HIGH^/RT^−^ and Bax^LOW^/RT^−^ (Fig. 3a–d). The prognostic significance of these subgroups was evaluated by Log-rank (Mantel Cox) test. The Bax^HIGH^/RT^+^ subgroup showed longer survival rates for both OS (P = 0.0143, HR = 0.2984) and RFS (P = 0.0143, HR = 0.2684) when compared with the Bax^LOW^/RT^+^ subgroup. Most strikingly, the median OS rate for Bax^HIGH^/RT^+^ is 66.43 months vs. Bax^LOW^/RT^+^ is 17.25 months. The median RFS rate for Bax^HIGH^/RT^+^ is 105.1 months vs. 11.99 months for Bax^LOW^/RT^+^ group. By comparison with above analyzed results on radiotherapy alone, the risk factor HR reduced from HR = 0.431 (RT^+^) to HR = 0.298 (Bax^HIGH^/RT^+^) for OS and from HR = 0.487 (RT^+^) to HR = 0.268 (Bax^HIGH^/RT^+^) for RFS. The median OS rate increased from 44.62 (RT^+^) to 66.43 months and median RFS increased from 39.36 months (RT^+^) to 105.1 months (Bax^HIGH^/RT^+^). This indicates that the levels of Bax protein have a great impact on the clinical outcome in patients underwent radiotherapy. The Bax^HIGH^/RT^−^ subgroup showed significantly longer survival rates for RFS (P = 0.0148, HR = 0.2339) but not statistically significant for OS (P = 0.0754, HR = 0.3519) when compared with the Bax^LOW^/RT^−^ subgroup. Although the patients underwent radiotherapy have significantly improved clinical outcomes (Fig. 2; Table 2), the patients with higher Bax without radiotherapy (Bax^HIGH^/RT^−^) have longer OS and RFS compared with patients with lower Bax^LOW^/RT^+^ subgroup (23.5 vs. 17.3 months) and (25.4 vs. 12 months), respectively (Table 2). This indicates that higher expression of Bax predicts longer time to recurrences of glioma patients without radiotherapy.

Totally 75 patients received chemotherapy (with TMZ) together with or without radiotherapy after curative resection. The patients with lower Bax expression showed significantly decreased OS (P < 0.0001, HR = 0.115) and RFS (P < 0.0001, HR = 0.064) (Fig. 3e, f). These results demonstrate that lower expression of Bax protein is associated with worse clinical outcome in glioma patients after radiotherapy and/or chemotherapy. Combination of Bax expression levels with radiotherapy or chemotherapy is a potent prognostic predictor of clinical outcome in patients with glioma.

## Discussion

The molecular basis of glioma patients with long-term survival has not been fully defined. In this study, we demonstrate that glioblastoma has decreased levels of Bax protein compared with low grade glioma. Lower Bax protein expression confers a worse clinical outcome in patients with gliomas after surgical resection and radiotherapy/chemotherapy.

Decreased Bax protein levels are associated with worse clinical outcomes in many cancers due to its pro-apoptotic function [14, 37–39]. In cancer cells, Bax is a short-lived protein and its protein levels are not consistent with the levels of mRNA [14, 19]. Lost p53 transcriptional activity in glioma tumor is also associated with decreased Bax protein expression [23]. Our results showed that Bax protein expression is lower in glioblastoma compared with grade II glioma which has an increased Bax expression than normal brain tissue. However, it is unclear why and how low-grade gliomas have increased Bax expression compared with the normal control. Based on our retrospective analysis, Bax mRNA levels in glioblastoma are significantly higher than those in normal samples. Because no available online data on Bax mRNA in low grade gliomas, we are not able to evaluate Bax mRNA levels in these tumors. The precise mechanism of decreased Bax protein levels in glioblastoma is not clear and may be due to increased Bax degradation activity and/or decreased p53 transcriptional activity in these high-grade tumor cells.

Bax activation is a key step in the intrinsic apoptotic pathway which is triggered by DNA damage induced by chemo- and/or radiotherapies or spontaneous apoptosis in cancer cells. Lower levels of Bax protein are associated with decreased spontaneous apoptosis of cancer cells and poor prognosis in patients with cancer [40, 41]. In this study, we used both continuous and categorical analyses to determine the impact of Bax protein levels on clinical outcomes in patients with gliomas. Our data demonstrate that lower expression of Bax is significantly associated with higher grade glioma and shorter OS/RFS in patients with glioma. The median OS or RFS for this cohort of glioma patients is 28 or 23 months, respectively. With higher Bax expression, the median OS reaches to 45 and the median RFS to 50 months. This indicates that the levels of Bax protein play an important role in glioma cell spontaneous apoptosis vs. tumor growth. Therefore, targeting the anti-Bcl-2 family of proteins will have great potential in controlling disease progression of this cancer.

Radiotherapy and chemotherapy are the mainstay of treatment for gliomas but glioblastoma still remains incurable due to resistance to treatment-induced apoptosis. Lower Bax expression is one of the resistance mechanisms of glioma cells to chemo- or radiotherapy [9, 42]. Reliable methods for predicting treatment responses and clinical outcomes would be great beneficial to cure this disease. Patients with higher levels of Bax protein in glioma tissue showed significantly prolonged OS and RFS after radiotherapy or chemotherapy. This indicates that the levels of Bax protein is crucial for the responses to radiotherapy and chemotherapy. It was reported that treatment with BH3 mimetic ABT-737 releases the pro-apoptotic Bax protein from its binding partner Bcl-2 and potently induces apoptotic cell death in glioblastoma cells in vitro and in vivo [43]. Combination of Bcl-2 inhibitors with radiotherapy/chemotherapy could overcome resistance of glioma cells to these treatment.

In summary, we demonstrate that Bax protein but not mRNA expression is decreased in glioblastoma tissue. Lower expression of Bax is associated with worse clinical outcome in patients with gliomas with radiotherapy and/or chemotherapy. We therefore propose that Bax protein expression is a reliable and independent prognostic marker for predicting responses of radiotherapy/chemotherapy and clinical outcomes in patients with gliomas.

## Electronic supplementary material

Below is the link to the electronic supplementary material.

Supplementary material 1 (PDF 257 KB)

Supplementary material 2 (DOCX 27 KB)
