# Supplementary material for: Lower expression of Bax predicts poor clinical outcome in patients with glioma after curative resection and radiotherapy/chemotherapy
Source: J Neurooncol. 2018 Nov 16;141(1):71–81. doi: 10.1007/s11060-018-03031-9 (PMC6341054; doi:10.1007/s11060-018-03031-9)
Supplement: Supplementary file 1 — Supplementary material 1 (PDF 257 KB) [file 11060_2018_3031_MOESM1_ESM.pdf]

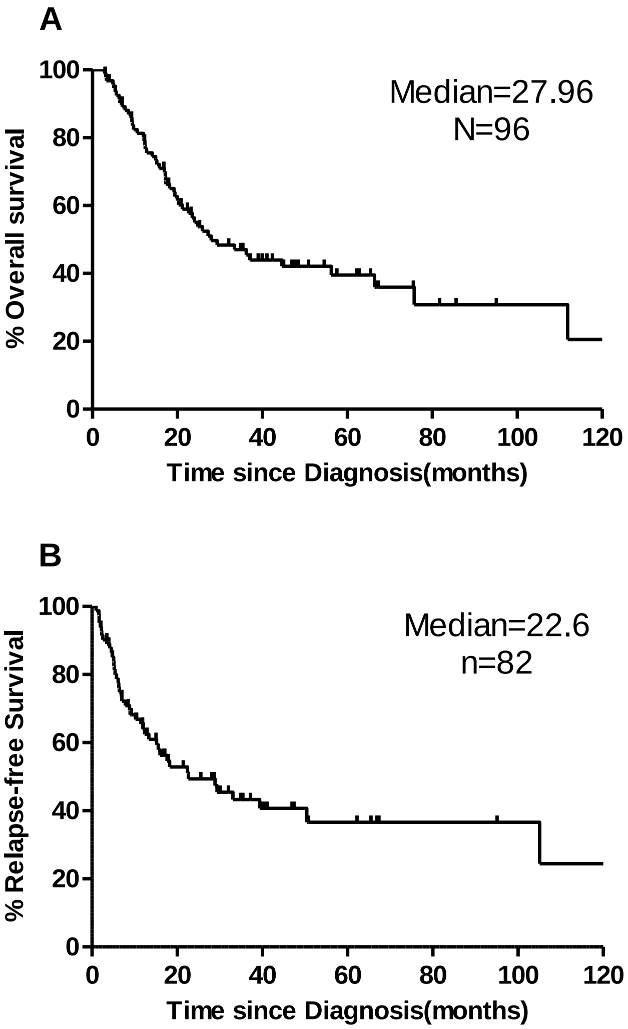

Suppl Figure 1. Kaplan-Meier survival curves for the entire cohort . (A) OS and (B) RFS survival curves were generated by GraphPad Prism software. 'Median' means the median survival time (months); 'n' indicates the numbers of patients.

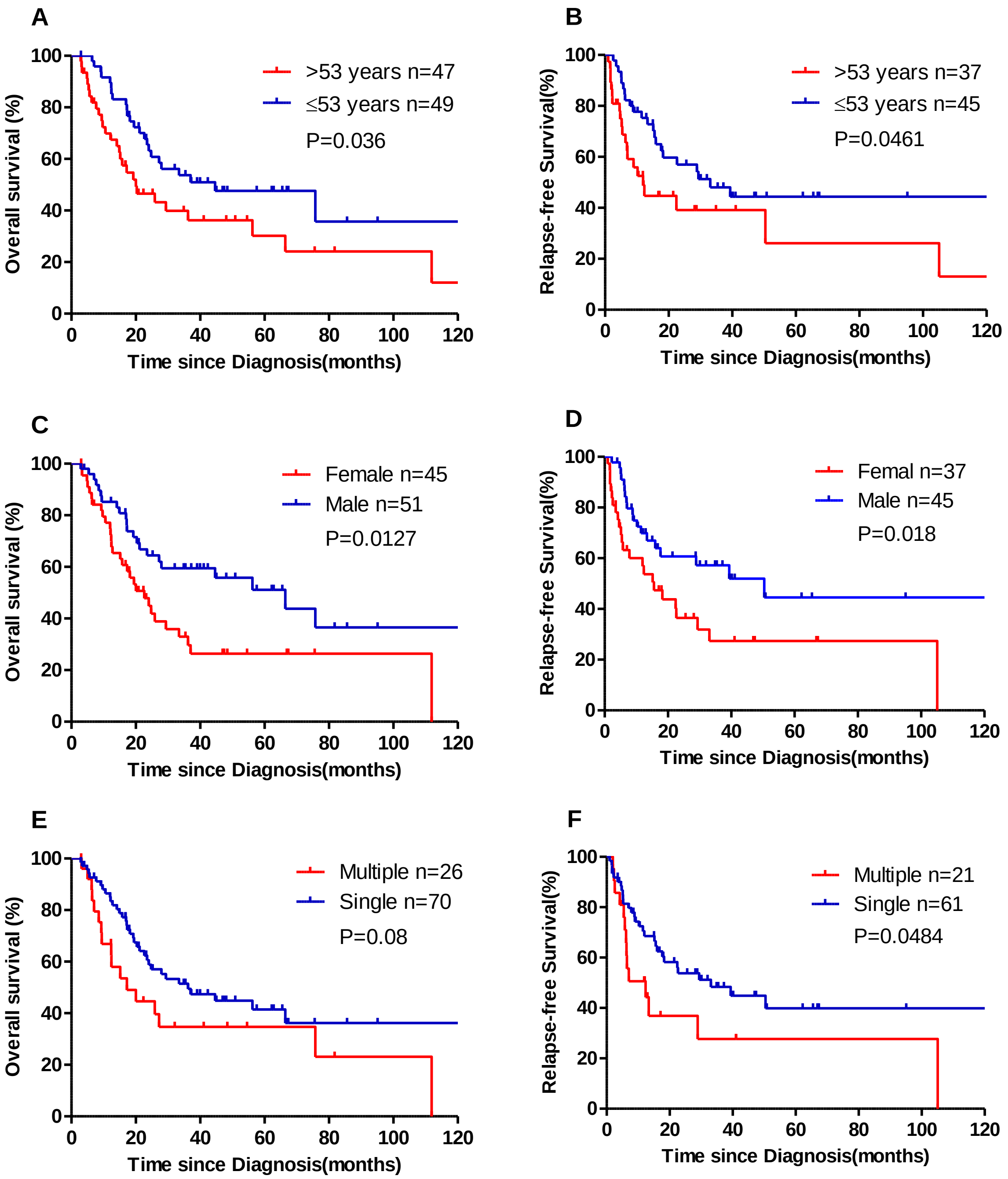

Suppl Figure 2. Kaplan-Meir curves of OS and RFS for each risk factor. Kaplan–Meier survival curves were generated using GraphPad Prism software. (A and B) OS and RFS of glioma patients on the basis of median age (>53 years/≤53 years) for entire cohort. (C and D) OS and RFS of glioma patients based on female or male for entire cohort. (E and F) OS and RFS on the basis of tumor number (multiple/single) for entire cohort. 'n', numbers of patients.

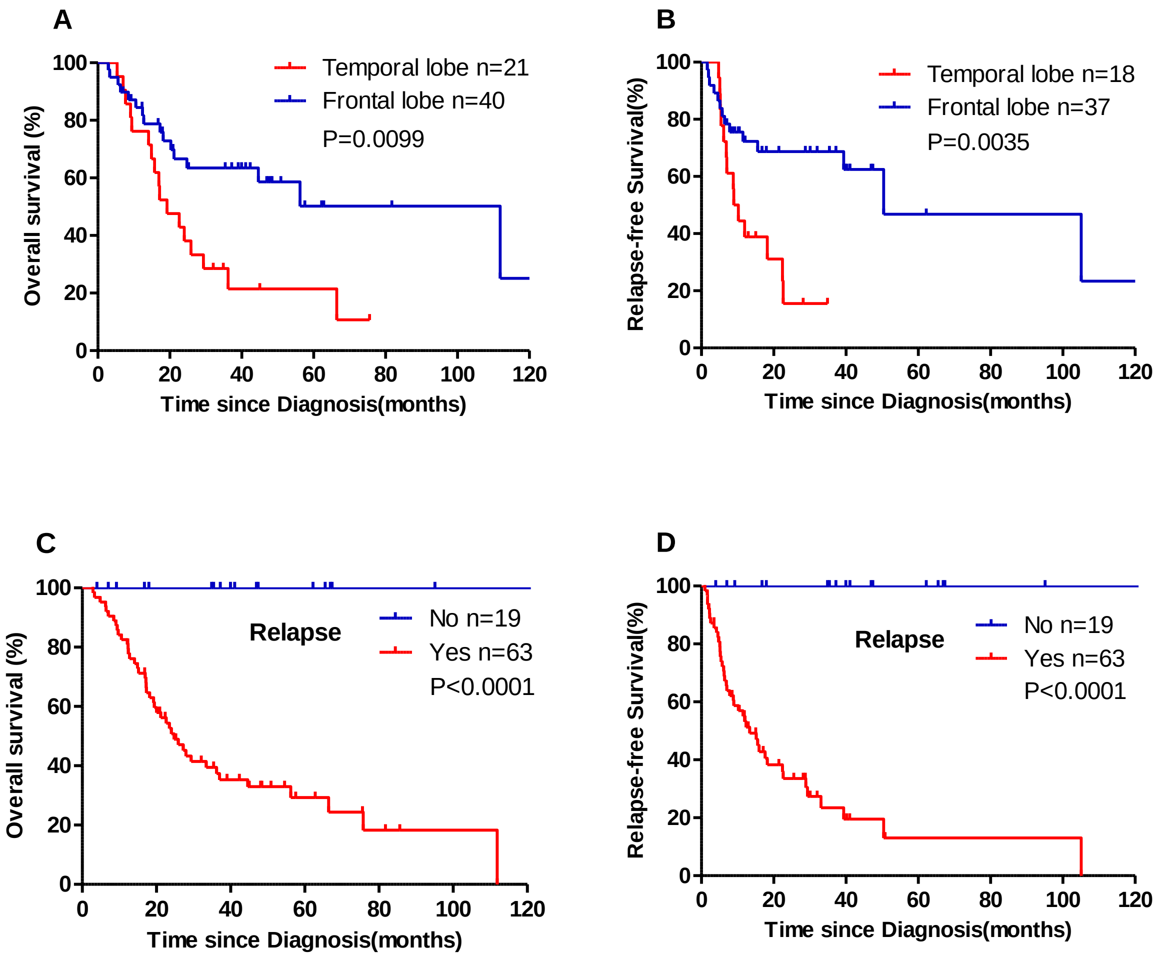

Suppl Figure 3. Kaplan-Meir curves of OS and RFS for tumour location and relapse. Kaplan–Meier survival curves were generated using GraphPad Prism software. (A and B) OS and RFS on the basis of tumour location (temporal/frontal). (C and D) OS and RFS of glioma patients on the basis of whether the tumours had relapse (yes/no). ‘n’, numbers of patients.

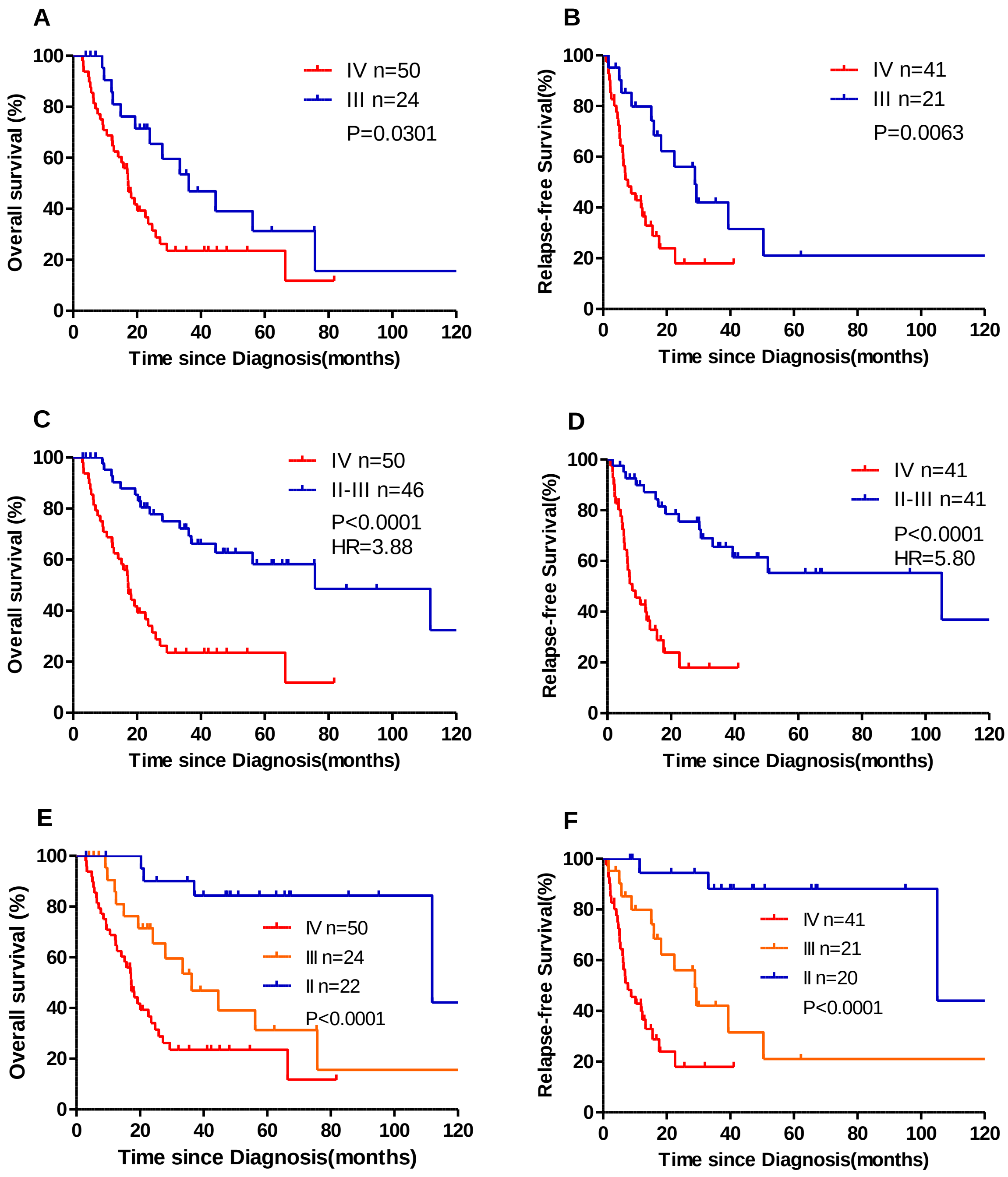

Suppl Figure 4. Kaplan-Meir curves of OS and PFS for WHO Grades. Kaplan–Meier survival curves were generated using GraphPad Prism software. (A and B) OS and RFS of glioma patients on the basis of WHO Grade (III/IV). (C and D) OS and RFS of glioma patients based on WHO II-III Grade or WHO IV Grade. (E and F) OS and RFS of glioma patients on the basis of WHO Grades (II/III/IV). ‘n’, numbers of patients.

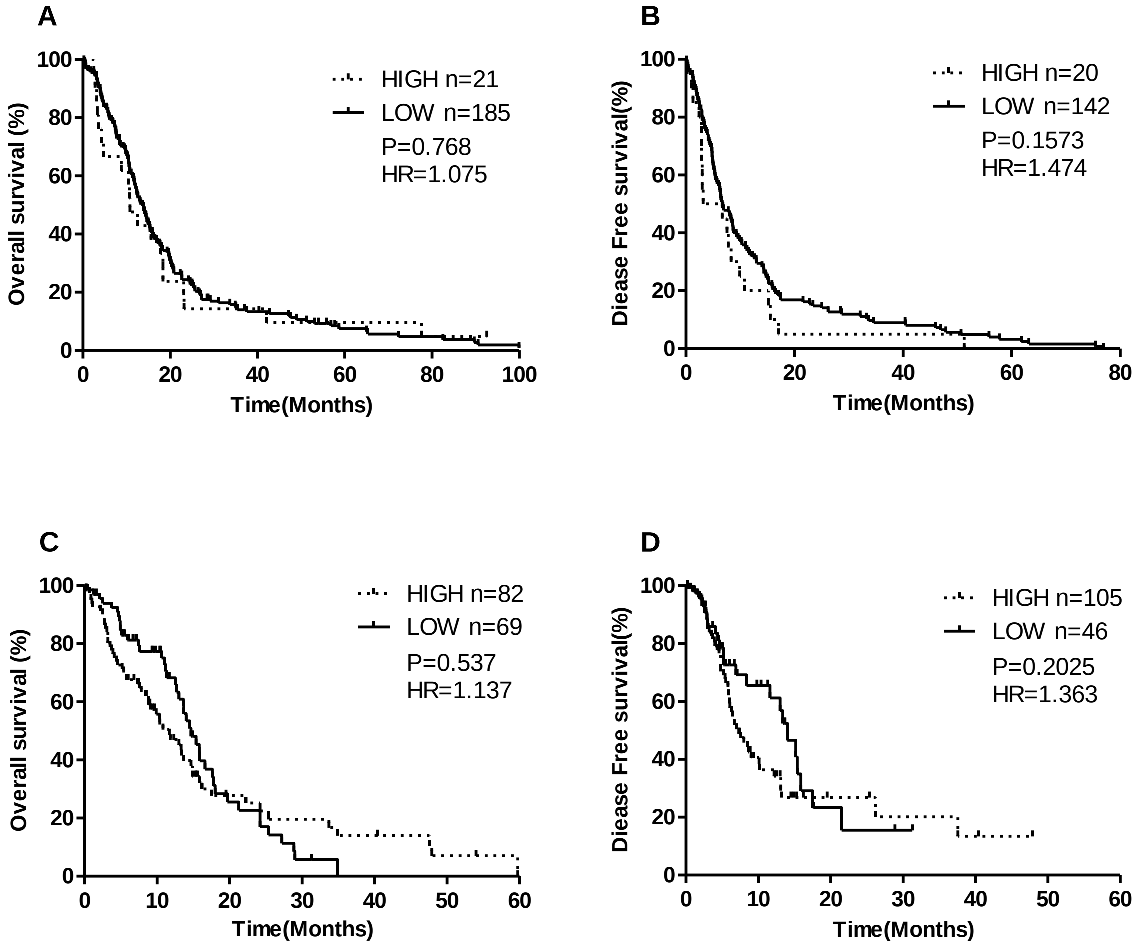

Suppl Figure 5. Categorical analysis of prognostic effect of Bax mRNA levels used online GBM data. Kaplan–Meier survival curves were generated using GraphPad Prism software. (A) OS of GBM patients was based on low (≤0.4906667) and high (>0.4906667) Bax mRNA levels for Nature BGM data. (B) RFS of glioma patients was based on low (≤0.4773333) and high (>0.4773333) Bax mRNA levels for Nature GBM data. (C) OS of GBM patients was based on low (≤1726) and high (>1726) Bax mRNA levels for Cell data. (D) RFS of GBM patients was based on low (≤1338) and high (>1338) Bax mRNA levels for Cell GBM data. The cut points were generated by the X-Tile software. 'n', numbers of patients.

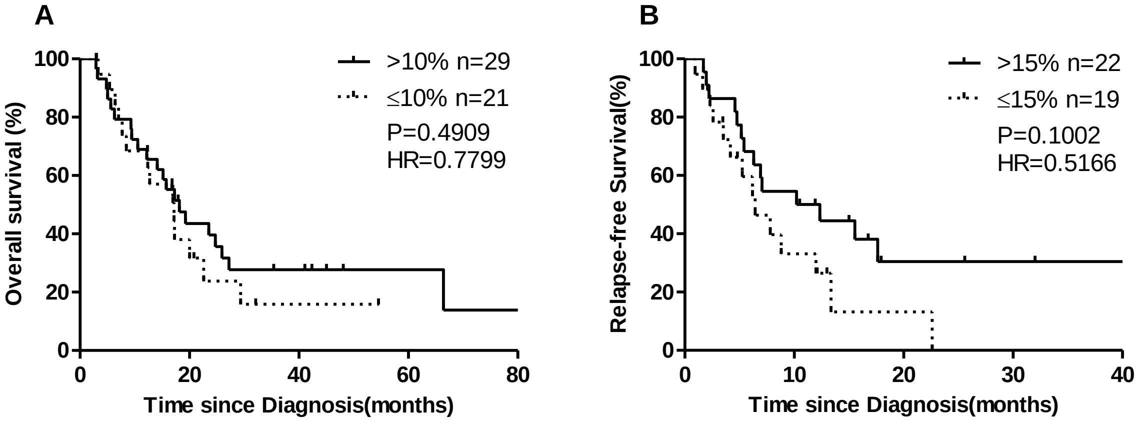

Suppl Figure 6. Stratification of Bax protein expression on prognosis in patients with glioblastoma. Kaplan–Meier survival curves were generated using GraphPad Prism software. (A) OS of glioblastoma patients was based on low (≤10%) and high (>10%) Bax expression levels for our data. (B) RFS of glioblastoma patients was based on low (≤15%) and high (>15%) Bax expression levels for our data. The cut points were generated by the X-Tile software. 'n', numbers of patients.

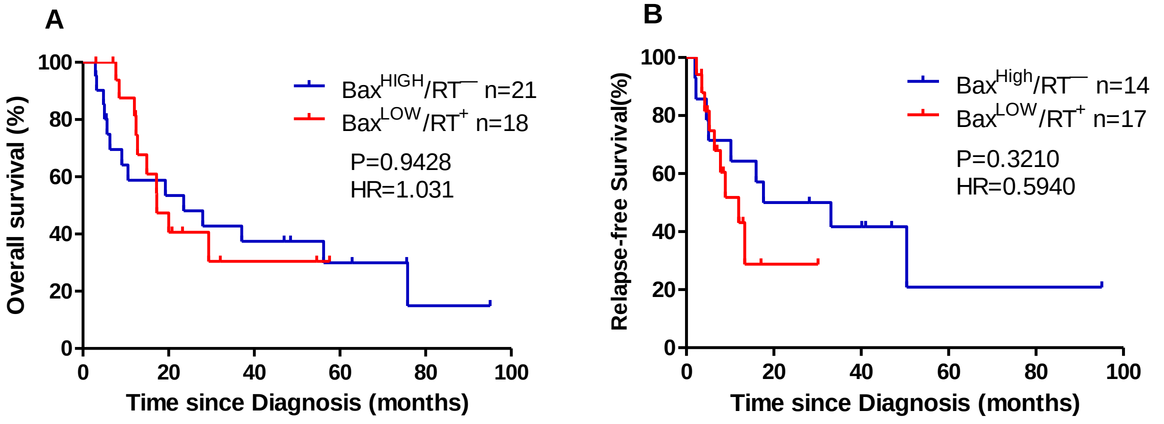

Suppl Figure 7. Kaplan-Meir curves of OS and RFS for Bax^{HIGH}/RT$^{—}$ and Bax^{LOW}/RT^{+} subgroup. (A) Overall survival. Two subgroups were defined according to the cut-off point for Bax (10%) and whether the patients had finished the radiotherapy: Bax^{HIGH}/RT$^{—}$= Bax >10% and the patients had not underwent or completed the radiotherapy; Bax^{LOW}/RT+ = Bax ≤10% and the patients had completed the radiotherapy. (B) Relapse-free survival. Two subgroups were defined according to the cut-off point for Bax (15%) and whether the patients had finished the radiotherapy: Bax^{HIGH}/RT$^{—}$ = Bax >15% and the patients had not underwent or completed the radiotherapy; Bax^{LOW}/RT^{+} = Bax ≤15% and the patients had finished the radiotherapy. The cut points were generated by the X-Tile software. 'n', numbers of patients.
