# Supplementary material for: Lower expression of Bax predicts poor clinical outcome in patients with glioma after curative resection and radiotherapy/chemotherapy
Source: J Neurooncol. 2018 Nov 16;141(1):71–81. doi: 10.1007/s11060-018-03031-9 (PMC6341054; doi:10.1007/s11060-018-03031-9)
Supplement: Supplementary file 2 — Supplementary material 2 (DOCX 27 KB) [file 11060_2018_3031_MOESM2_ESM.docx]

**Suppl Table 1. Patient Demographics and Clinical Characteristics**

| **Factors** | **No of Patients (%)** |
| --- | --- |
| **Total No of Subject** | 96 (100%) |
| **Age (Y)** |  |
| Median | 53 |
| Range | 2-80 |
| **Gender** |  |
| Male | 51 (53) |
| Female | 45 (47) |
| **Tumour Number** |  |
| Signal | 70 (73) |
| Multiple | 26 (27) |
| **Tumour Location** |  |
| Frontal | 40 (41) |
| Temporal | 21 (22) |
| Others | 35 (36) |
| **WHO Grade** |  |
| II | 22 (24) |
| III | 24 (25) |
| IV | 50 (51) |
| **Relapse** |  |
| Yes | 64(67) |
| No | 18(19) |
| Non-specific | 14(14) |
| **Radiotherapy** |  |
| Yes | 66(69) |
| No | 30(31) |
| **Chemotherapy** |  |
| Yes | 74(77) |
| No | 22(33) |

**Suppl Table 2. Survival characteristics for glioma patients**

|  | **All patients** |
| --- | --- |
| **Overall survival (months)** | **N=96** |
| **Events (%)** | 52 (45.8%) |
| **Median follow up (95% CI)** | 27.96 (15.34-40.58) |
| **Relapse-free survival (Months)** | **n=82** |
| **Events (%)** | 39 (43.6%) |
| **Median follow up (95% CI)** | 22.6 (7.04-38.17) |

**Suppl Table 3. Confounders of continuous covariates on clinical outcome of glioma**

| **Confounding effects** | **OS** | **PFS** |
| --- | --- | --- |
| **Bax** | —— | —— |
| **Age** | 1.3% | 0.8% |
| **WHO Grade** | 10.4%* | 10.4% |

**Note: * The confounding effect was calculated according to the formula = (HR_crude_- HR_adjusted_)/HR_crude_ X 100%. The value > 10% is defined as having a confounding effect.**

**Suppl Table 4. Association of Bax expression with patients’ clinicopathological features in gliomas.**

| Variable | All cases | Low  expression | High  expression | p Value* |
| --- | --- | --- | --- | --- |
| **Age (years)** |  |  |  | 0.419 |
| ≤53† | 49 | 12(24.5%) | 37(75.5%) |  |
| >53 | 47 | 15(31.9%) | 32(68.1%) |  |
| **Gender** |  |  |  | 0.286 |
| Male | 51 | 12(23.5%) | 39(76.5%) |  |
| Female | 45 | 15(33.3%) | 30(66.7%) |  |
| **Tumour number** |  |  |  | 0.389 |
| Signal | 70 | 18(25.7%) | 52(74.3%) |  |
| Mutiple | 26 | 9(34.6%) | 17(65.4%) |  |
| **Tumour location** |  |  |  | 0.032 |
| Frontal | 40 | 7(17.5%) | 33(82.5%) |  |
| Temporal | 21 | 9(42.9%) | 12(57.1%) |  |
| **WHO Grade** |  |  |  | 0.003 |
| II | 22 | 1(4.5%) | 21(95.5%) |  |
| III | 24 | 5(20.8%) | 19(79.2%) |  |
| IV | 50 | 21(42.0%) | 29(58.0%) |  |
| **Relapse** |  |  |  | 0.027 |
| Yes | 64 | 20(31.3%) | 44(68.8%) |  |
| No | 18 | 1(5.6%) | 17(94.4%) |  |
| **Radiotherapy** |  |  |  | 0.783 |
| Yes | 66 | 18(27.3%) | 48(72.7%) |  |
| No | 30 | 9(30.0%) | 21(70.0%) |  |
| **Chemotherapy** |  |  |  | 0.208 |
| Yes | 74 | 24(32.4%) | 51(68.9%) |  |
| No | 22 | 4 (18.2%) | 18(81.8%) |  |

***Chi-square test.**

**†Median age.**

**Suppl Table 5. Continuous analysis of the prognostic effect of Bax mRNA levels.**

| **Bax mRNA** | **OS** | | **DFS** | |
| --- | --- | --- | --- | --- |
|  | **HR** | ***P*** | **HR** | ***P*** |
| **Nature Data** | 1.014(0.751-1.379) | 0.927 | 1.398(1.000-1.954) | 0.050 |
| **Cell Data** | 1.00(1.00-1.00) | 0.88 | 1.00(1.00-1.00) | 0.61 |

**Suppl Table 6. Continuous analysis of the prognostic effect of Bax expression levels.**

| **Bax protein** | **OS** | | **DFS** | |
| --- | --- | --- | --- | --- |
|  | **HR** | ***P*** | **HR** | ***P*** |
| **Our Data** | 0.786(0.395-1.564) | 0.492 | 0.535(0.251-1.140) | 0.105 |
